# Detective flow imaging-assisted endoscopic ultrasound-guided radiofrequency ablation of hepatocellular carcinoma adjacent to the inferior vena cava

**DOI:** 10.1055/a-2954-0511

**Published:** 2026-09-28

**Authors:** Gianenrico Rizzatti, Giulia Palumbo, Giulia Tripodi, Fabrizio Termite, Leonardo Stella, Francesca Romana Ponziani, Cristiano Spada

**Affiliations:** 1 Digestive Endoscopy Unit 18654 Fondazione Policlinico Universitario A. Gemelli, IRCCS – Università Cattolica del Sacro CuoreRomeLazioItaly; 2 Liver Unit, Centro Malattie dell'Apparato Digerente (CEMAD) 18654 Fondazione Policlinico Universitario A. Gemelli, IRCCS – Università Cattolica del Sacro CuoreRomeLazioItaly

An 83-year-old man with a history of orthotopic liver transplantation developed an 18-mm hepatocellular carcinoma (HCC) in segment I during surveillance. Due to close proximity of the lesion to the caval vein, percutaneous ablation was considered technically challenging, with an increased risk of incomplete treatment and early recurrence related to the “heat sink” effect caused by blood flow-mediated heat dissipation. 1​

Given these considerations, endoscopic ultrasound-guided radiofrequency ablation (EUS-RFA) was performed with a therapeutic linear-array echoendoscope. EUS-RFA was performed with a 19G electrode needle, a dedicated generator, and a cooling system. The electrode was positioned in the central portion of the lesion, and radiofrequency was delivered until a significant increase in impedance was detected. 2​

The identification of residual vital tissue during EUS-RFA represents a major challenge. Administration of intravenous contrast agents has been proposed to assess residual vascularized areas; however, its use is impaired by limited window time and potential adverse events. Detective flow imaging (DFI), a novel advanced Doppler technique, enables the real-time visualization of low-velocity microvascular flow without contrast administration. DFI was used throughout the procedure to continuously assess the presence of residual vital tissue especially in the part adjacent to the inferior vena cava, where the risk of incomplete ablation due to the heat sink effect was increased ( Fig. 1 ). DFI allowed the accurate targeting of residual vascularized areas, including previously treated regions where visibility was impaired by treatment-related artifacts ( Video 1 ).

**Fig. 1:**
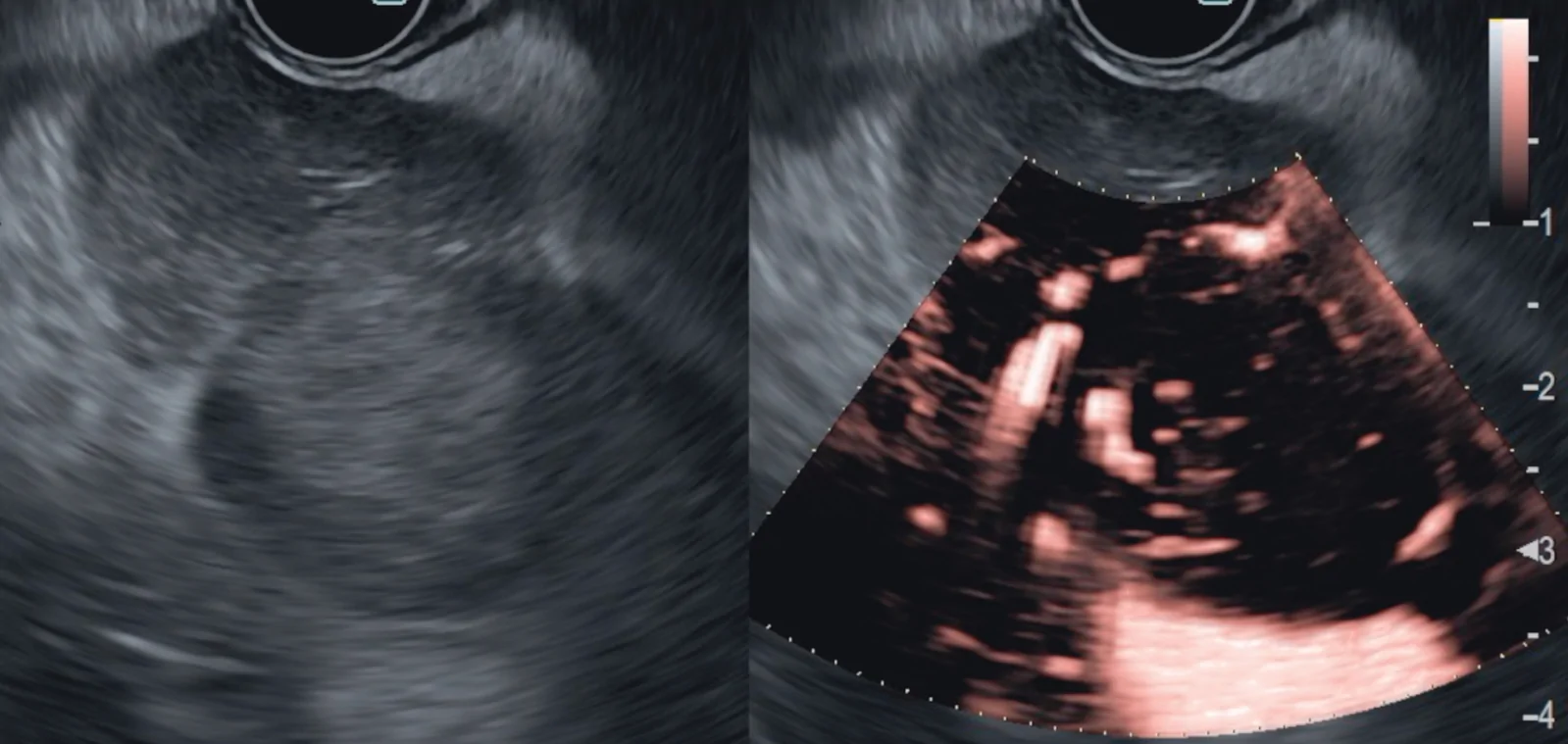
Endoscopic ultrasound with DFI demonstrating microvascular flow within hepatocellular carcinoma adjacent to the inferior vena cava. DFI, detective flow imaging.

**Video 1** EUS-guided radiofrequency ablation (EUS-RFA) for a caudate lobe hepatocellular carcinoma adjacent to the inferior vena cava. The application of detective flow imaging (DFI) enabled the real-time visualization of microvascular flow, optimizing ablation precision.


Contrast-enhanced computed tomography performed one month after EUS-RFA demonstrated complete tumor ablation without evidence of residual disease.

This case highlights the potential role of therapeutic EUS in anatomically challenging HCC locations. The adjunctive use of DFI may improve the precision and safety of EUS-guided ablation without contrast agents, supporting its potential role in advanced interventional endoscopy.

Endoscopy_UCTN_Code_TTT_1AS_2AH
